# Outcome reporting across randomized controlled trials evaluating potential treatments for male infertility: a systematic review

**DOI:** 10.1093/hropen/hoac010

**Published:** 2022-03-04

**Authors:** Michael P Rimmer, Ruth A Howie, Venkatesh Subramanian, Richard A Anderson, Ricardo Pimenta Bertolla, Yusuf Beebeejaun, Pietro Bortoletto, Sesh K Sunkara, Rod T Mitchell, Allan Pacey, Madelon van Wely, Cindy M Farquhar, James M N Duffy, Craig Niederberger

**Affiliations:** MRC Centre for Reproductive Health, Queens Medical research Institute, University of Edinburgh, Edinburgh, UK; Edinburgh Fertility Centre, Simpsons Centre for Reproductive Health, Royal Infirmary of Edinburgh, Edinburgh, UK; King’s Fertility, The Fetal Medicine Research Unit, King’s College London, London, UK; MRC Centre for Reproductive Health, Queens Medical research Institute, University of Edinburgh, Edinburgh, UK; Edinburgh Fertility Centre, Simpsons Centre for Reproductive Health, Royal Infirmary of Edinburgh, Edinburgh, UK; Division of Urology, Department of Surgery, Universidade Federal de Sao Paulo, São Paulo, Brazil; King’s Fertility, The Fetal Medicine Research Unit, King’s College London, London, UK; The Ronald O. Perelman and Claudia Cohen Center for Reproductive Medicine, Weill Cornell Medicine, New York, NY, USA; Division of Women’s Health, Faculty of Life Sciences and Medicine, King’s College London, London, UK; MRC Centre for Reproductive Health, Queens Medical research Institute, University of Edinburgh, Edinburgh, UK; Department of Oncology and Metabolism, University of Sheffield, Sheffield, UK; Amsterdam University Medical Centers, Amsterdam, The Netherlands; Cochrane Gynaecology and Fertility Group, Auckland, New Zealand; Department of Obstetrics and Gynaecology, University of Auckland, Auckland, New Zealand; King’s Fertility, The Fetal Medicine Research Unit, King’s College London, London, UK; Department of Urology, University of Illinois at Chicago, Chicago, IL, USA; Department of Bioengineering, University of Illinois at Chicago College of Engineering, Chicago, IL, USA

**Keywords:** clinical practice guidelines, core outcome set, male infertility, outcome reporting, randomized controlled trials, systematic review

## Abstract

**STUDY QUESTION:**

What are the primary outcomes and outcome measures used in randomized controlled trials (RCTs) evaluating potential treatments for male infertility in the last 10 years?

**SUMMARY ANSWER:**

Outcome reporting across male infertility trials is heterogeneous with numerous definitions and measures used to define similar outcomes.

**WHAT IS KNOWN ALREADY:**

No core outcome set for male infertility trials has been developed. Male infertility trials are unique in that they have potentially three participants, a man, a female partner and their offspring and this will likely lead to significant variation in outcome reporting in randomized trials.

**STUDY DESIGN, SIZE, DURATION:**

A systematic review of RCTs mapping outcomes and outcome measures evaluating potential treatments for men with infertility registered in the Cochrane Register of Controlled Trials (CENTRAL) between January 2010 and July 2021.

**PARTICIPANTS/MATERIALS, SETTING, METHODS:**

Abstract screening and study selection was undertaken in duplicate using a review protocol that was developed prior to commencing the review. No risk of bias assessment was undertaken as this review aims to report on outcome reporting only.

**MAIN RESULTS AND THE ROLE OF CHANCE:**

One hundred and seventy-five RCTs were identified, and given the large number of studies we limited our review to the 100 largest trials. Seventy-nine different treatments were reported across the 100 largest RCTs including vitamin and dietary supplements (18 trials), surgical treatments (18 trials) and sperm selection techniques (22 trials). When considering the largest 100 trials (range: 80–2772 participants), 36 primary and 89 secondary outcomes were reported. Forty-seven trials reported a primary outcome and 36 trials clearly defined their primary outcome. Pregnancy outcomes were inconsistently reported and included pregnancy rate (51 trials), pregnancy loss including miscarriage, ectopic pregnancy, stillbirth (9 trials) and live birth (13 trials). Trials consistently reporting the same outcome frequently used different definitions. For example, semen quality was reported by 75 trials and was defined in 7 different ways, including; the World Health Organization (WHO) 2010 criteria (32 trials), WHO 1999 criteria (18 trials), WHO 1992 criteria (3 trials), WHO 1999 and 1992 criteria (1 trial) and the Kruger strict morphology criteria (1 trial).

**LIMITATIONS, REASONS FOR CAUTION:**

We only evaluated the 100 largest trials published in the last 10 years and did not report outcomes on the remaining 75. An outcome was included as a primary outcome only if clearly stated in the manuscript and we did not contact authors to clarify this. As our review mapped outcomes and outcome measures, we did not undertake an integrity assessment of the trials included in our review.

**WIDER IMPLICATIONS OF THE FINDINGS:**

Most randomized trials evaluating treatments for male infertility report different outcomes. Only half of the RCTs reported pregnancy rate and even fewer reported live birth; furthermore, the definitions of these outcomes varies across trials. Developing, disseminating and implementing a minimum data set, known as a core outcome set, for male infertility research could help to improve outcome selection, collection and reporting.

**STUDY FUNDING/COMPETING INTEREST(S):**

A.P.—chairman of external scientific advisory committee of Cryos International Denmark ApS, member of the scientific advisory board for Cytoswim LDT and ExSeed Health. Guest lecture at the ‘Insights for Fertility Conference’, funded by MERK SERONO Limited. M.v.W.—holds a ZON-MW research grant. No external funding was obtained for this study.

WHAT DOES THIS MEAN FOR PATIENTS?

This study looks at what information randomized controlled trials (RCTs) collect and report, to help evaluate possible treatments for male infertility.

Male infertility affects millions of men worldwide, and many different treatments have been proposed for this. Treatments with the potential to reduce this health burden require robust evaluation. When assessing new treatments, RCTs are considered the ‘gold-standard’ method. How effective these treatments are can only be truly understood if clinical trials report the same outcomes, which are measured and defined in the same way.

We identified many RCTs that reported different outcomes, for example semen parameters, pregnancy rate or live birth, making it challenging to combine the results of these trials. Even when trials did report the same outcome, for example pregnancy rate, the outcome was either undefined or defined in numerous different ways. This means that when new RCTs are published to evaluate a treatment for male infertility, researchers and clinicians may not be able to truly understand its potential benefit for patients, in the context of previously published research.

## Introduction

Infertility affects 50 million couples globally (Martinez *et al.*, 2012; Vander Borght and Wyns, 2018). Male factor infertility affects up to 18 million men worldwide (Winters and Walsh, 2014; Agarwal *et al.*, 2015) and is recognized as a contributing factor in up to one-third of cases (Thonneau *et al.*, 1991; Agarwal *et al.*, 2015; Tamrakar and Bastakoti, 2019). Treatments with the potential to reduce this health burden require robust evaluation. When assessing new treatments, randomized controlled trials (RCTs) are considered the gold-standard method to determine the efficacy and safety of potential treatments (Liberati *et al.*, 2009). However, despite their potentially robust design, methodology and conduct, RCTs are only as meaningful as the outcomes they collect and report (Ioannidis *et al.*, 2014; Duffy *et al.*, 2019).

Complex issues, including a failure to consider the perspectives of people with fertility problems when selecting outcomes, variations in outcome definitions and measurement instruments as well as outcome reporting bias can make the selection, collection and reporting of outcomes challenging. The unique nature of male infertility research can add further complexity as outcomes will often need to consider three research participants, namely the male, his female partner or gestational carrier, and their subsequent offspring.

Little is known about outcome reporting in male infertility clinical trials. To understand the heterogeneity in outcome reporting of RCTs in male infertility, and provide a basis for more consistent reporting to the highest possible standards, we undertook a systematic review of the outcomes and outcome measures reported by the 100 largest RCTs published over the last 10 years. Reporting on the outcomes, outcome measures and consistency of these outcomes across trials will enable us to identify how outcome reporting could be standardized in future trials. This will allow researchers to better understand the true efficacy of interventions assessed in RCTs to address male infertility.

## Materials and methods

A protocol was developed prior to commencing the review and included clearly defined objectives, including search criteria, study selection criteria and extraction of data (Supplementary Data). We followed the reporting guidelines for systematic reviews of RCTs, as outlined by the Preferred Reporting Items for Systematic Reviews and Meta-Analyses (PRISMA) statement (Minebois *et al.*, 2017).

The objective of our review was to characterize outcome reporting across RCTs evaluating interventions for male infertility. Our main outcome of interest was primary outcome reporting in these trails and the definition of this outcome. RCTs were identified by searching the Cochrane Register of Controlled Trials (CENTRAL) for RCTs published between 1 January 2010 and 24 July 2021. CENTRAL is populated by the Cochrane Collaboration by regularly searching the Cumulative Index to Nursing and Allied Health Literature (CINAHL), EMBASE, MEDLINE and PsycINFO (Supplementary Data). Two authors (M.P.R. and R.A.H.) independently performed the screening of each potentially relevant record, based on the title and abstract and reviewed the full text of each selected study to assess eligibility. Where data were reported twice, such as a conference abstract and peer reviewed paper published at a later date, extracted data from the peer reviewed paper was used. Discrepancies between the authors were resolved through discussion and a consensus being agreed.

We included all RCTs which evaluated potential treatments for male factor infertility. We excluded systematic reviews and non-randomized trials. We limited our search to publications written in English. The largest 100 RCTs based on the number of participants were included in our analysis.

Using a standardized data extraction form, two authors (M.P.R. and R.A.H.) independently extracted study characteristics, nature of the intervention and both the primary and secondary outcomes reported. We reported the definitions used for commonly reported outcomes, including semen quality, pregnancy rate and live birth, to illustrate how these definitions varied. An outcome was considered to be a primary outcome only if this was clearly started in the method section. Discrepancies between authors were resolved through discussion and a consensus being achieved. A comprehensive inventory of outcomes was developed. We used descriptive statistics to characterize outcome reporting across included RCTs. No risk of bias was undertaken as the scope of this review was to report outcome reporting across RCTs and not to assess the quality of the trials.

## Results

**Figure 1.**
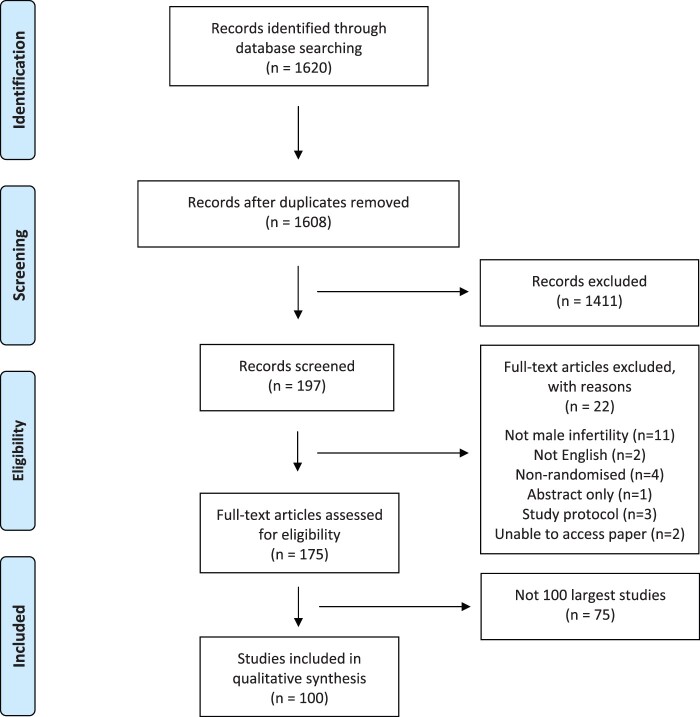
**PRISMA flow diagram outlining number of studies identified from our database search, number remaining after screening and the number of studies reporting interventions for male infertility included in our analysis.** PRISMA, Preferred Reporting Items for Systematic Reviews and Meta‐Analyses.

We identified 1620 records. After excluding 12 duplicate records, 1608 titles and abstracts were screened to identify RCTs evaluating interventions for male infertility (Fig. 1). We excluded 1411 records as they were either non-randomized studies, systematic reviews, did not report an intervention for male infertility or did not report on a male infertility cohort. Two independent reviewers evaluated the remaining 197 potentially relevant trials of which 175 were deemed to be relevant. From these, the largest 100 RCTs reporting data from 24 542 men (range: 80–2772 men) were used to identify and report outcomes (Abdel-Maguid and Othman, 2010; Fang *et al.*, 2010; Fayez *et al.*, 2010; Kovacic *et al.*, 2010; Abdel-Meguid *et al.*, 2011; Azadi *et al.*, 2011; Balaban *et al.*, 2011; Figueira Rde *et al.*, 2011; Hafeez *et al.*, 2011; Safarinejad, 2011; Safarinejad *et al.*, 2011; Selice *et al.*, 2011; Turhan *et al.*, 2011; Wilding *et al.*, 2011; Amirzargar *et al.*, 2012; Colacurci *et al.*, 2012; El-Khayat *et al.*, 2012; Lee *et al.*, 2012; Mansour Ghanaie *et al.*, 2012; Parmegiani *et al.*, 2012; Rago *et al.*, 2012; Safarinejad *et al.*, 2012; Velaers *et al.*, 2012; Azizollahi *et al.*, 2013; De Vos *et al.*, 2013; Gopinath *et al.*, 2013; Kang *et al.*, 2013; Leandri *et al.*, 2013; Majumdar and Majumdar, 2013; Pan *et al.*, 2013; Worrilow *et al.*, 2013; Akin *et al.*, 2014; Karamahmutoglu *et al.*, 2014; Kolahdooz *et al.*, 2014; Moslemi Mehni *et al.*, 2014; Nematollahi-Mahani *et al.*, 2014; Pourmand *et al.*, 2014; Raigani *et al.*, 2014; Romany *et al.*, 2014; Wang *et al.*, 2014; Calogero *et al.*, 2015; Cyrus *et al.*, 2015; Ding *et al.*, 2015; ElSheikh *et al.*, 2015; Farrag *et al.*, 2015; Guo *et al.*, 2015; Haje and Naoom, 2015; Hou *et al.*, 2015; Peivandi *et al.*, 2015; Sikka *et al.*, 2015; Youssef and Abdalla, 2015; Hosseini *et al.*, 2016; Jin *et al.*, 2016; Nasr Esfahani *et al.*, 2016; Pan *et al.*, 2016; Park *et al.*, 2016; Bryniarski *et al.*, 2017; Guo *et al.*, 2017; Milardi *et al.*, 2017; Qu *et al.*, 2017; Rosety *et al.*, 2017; Taiyeb *et al.*, 2017; Hajizadeh Maleki and Tartibian, 2017a,b; Babak *et al.*, 2018; Blomberg Jensen *et al.*, 2018; Bodin *et al.*, 2018; Busetto *et al.*, 2018; Habous *et al.*, 2018; Hajizadeh Maleki and Tartibian, 2018; Ketabchi and Salajegheh, 2018; Ketabchi *et al.*, 2018; Nasimi Doost Azgomi *et al.*, 2018; Sun *et al.*, 2018; Tsounapi *et al.*, 2018; Almekaty *et al.*, 2019; Chen *et al.*, 2019; De Geyter *et al.*, 2019; Hajizadeh Maleki *et al.*, 2019; Kızılay and Altay, 2019; Lin *et al.*, 2019; Mangoli *et al.*, 2019; Miller *et al.*, 2019; Tehrani *et al.*, 2019; Yetkinel *et al.*, 2019; Yu *et al.*, 2019; Zhao *et al.*, 2019; Chen *et al.*, 2020; Degirmenci *et al.*, 2020; Eslamian *et al.*, 2020; Hajizadeh Maleki and Tartibian, 2020; Hasanen *et al.*, 2020; Huang *et al.*, 2020; Joseph *et al.*, 2020; Karimi *et al.*, 2020; Kopets *et al.*, 2020; Liu *et al.*, 2020; Schisterman *et al.*, 2020; Bozhedomov *et al.*, 2021; Salas-Huetos *et al.*, 2021).

Seventy-nine different treatments were reported across the 100 RCTs (Table I). These included trials reporting vitamin or dietary supplements or nutraceuticals (n = 18), surgical procedures (n = 18) and sperm selection or modification techniques (n = 22).

**Table I:** Characteristics of the 100 largest trials included in this review evaluating interventions for male infertility.

Study	Intervention group one	Intervention group two	Participants (n)
Miller *et al.* (2019)	Physiological intracytoplasmic sperm injection	Intracytoplasmic sperm injection	2772
Schisterman *et al.* (2020)	Folic acid and zinc sulphate	Placebo	2370
Worrilow *et al.* (2013)	Hyaluronic binding prior to intracytoplasmic sperm injection	Intracytoplasmic sperm injection	802
Huang *et al.* (2020)	Folic acid	Placebo	769
Kovacic *et al.* (2010)	Embryo culture in 5% oxygen	Embryo culture in 20% oxygen	647
Hajizadeh Maleki and Tartibian (2017a)	Exercise	No intervention	556
Hajizadeh Maleki and Tartibian (2020)	Exercise	No intervention	441
Hajizadeh Maleki and Tartibian (2018)	Exercise	No intervention	430
Hajizadeh Maleki and Tartibian(2017b)	Exercise	No intervention	419
Hasanen *et al.* (2020)	Physiological intracytoplasmic sperm injection	Magnetic activated cell sorting	413
Sun *et al.* (2018)	Bilateral varicocelectomy	Unilateral varicocelectomy	358
Ding *et al.* (2015) *	Recombinant FSH	Sodium chloride injection	354
Turhan *et al.* (2011)	Double sperm wash	Single sperm wash	341
De Vos *et al.* (2013)	Intracytoplasmic morphologically selected sperm injection	Intracytoplasmic sperm injection	340
Chen *et al.* (2019) *	Chymotrypsin treatment	Vitamin C, E, zinc gluconate and a spermatogenic tablet	337
Almekaty *et al.* (2019)	Artery preserving varicocelectomy	Artery ligating varicocelectomy	330
Blomberg Jensen *et al.* (2018)	Vitamin D and calcium	Placebo	330
Zhao *et al.* (2019)	hCG and hMG	Placebo	316
Velaers *et al.* (2012)	Single touch sperm immobilization	Triple touch sperm immobilization	290
Hajizadeh Maleki *et al.* (2019) *	Exercise	No intervention	283
Habous *et al.* (2018) *	Clomiphene citrate	hCG injections	282
Romany *et al.* (2014)	Sperm swim up and removal of annexin V positive sperm	Sperm swim up	263
Safarinejad *et al.* (2011)	Saffron	Placebo	260
Leandri *et al.* (2013)	Intracytoplasmic morphologically selected sperm injection	Intracytoplasmic sperm injection	255
Safarinejad (2011)	Pentoxifylline	Placebo	254
Wilding *et al.* (2011)	Intracytoplasmic morphologically selected sperm injection	Intracytoplasmic sperm injection	250
Taiyeb *et al.* (2017)	Prednisolone	Placebo	241
Moslemi Mehni *et al.* (2014 ) *	Pentoxifylline and l-carnitine	Placebo	235
Bodin *et al.* (2018)	Counselling	No intervention	229
Safarinejad *et al.* (2012)	Oral ubiquinol	Placebo	228
Karamahmutoglu *et al.* (2014)	Density gradient centrifugation	Swim up sperm preparation	223
Sikka *et al.* (2015)	Pregabalin	Placebo	222
Karimi *et al.* (2020)	Density gradient centrifugation and zeta selection	Density gradient centrifugation	220
Lin *et al.* (2019)	GnRH	hCG and hMG	220
Fang *et al.* (2010) *	Spermatic vein ligation, vitamin E, pentoxyfylline and clomiphene	Vitamin E, pentoxyfylline and clomiphene	219
Tsounapi *et al.* (2018) *	Phosphodiesterase type-5 inhibitor	No intervention	217
Rago *et al.* (2012)	Vardenafil	No intervention	205
Nasr Esfahani *et al.* (2016)	Density gradient centrifugation and zeta selection	Density gradient centrifugation	203
Joseph *et al.* (2020)	Vitamin C, Vitamin E, Zing	No antioxidants	200
Calogero *et al.* (2015)	Myoinositol and folic acid	Folic acid	194
Babak *et al.* (2018)	Varicocelectomy and hCG	Varicocelectomy	193
Guo *et al.* (2015)	Doppler ultrasound assisted subinguinal microscopic varicocelectomy	Microscopic varicocelectomy	180
Eslamian *et al.* (2020)	DHA vitamin, Vitamin E	Placebo	180
Balaban *et al.* (2011)	Intracytoplasmic morphologically selected sperm injection	Intracytoplasmic sperm injection	168
Abdel-Maguid and Othman (2010)	Microsurgical subinguinal varicocelectomy	Subinguinal varicocelectomy	162
Bozhedomov *et al.* (2021)	Hydrophilic nutrients	Lipophilic nutrients	160
Nematollahi-Mahani *et al.* (2014) *	Zinc sulphate and folic acid	Placebo	160
Azizollahi *et al.* (2013) *	Varicocelectomy and zinc sulphate	Varicocelectomy and placebo	160
Majumdar and Majumdar (2013)	Physiological intracytoplasmic sperm injection	Intracytoplasmic sperm injection	156
Fayez *et al.* (2010) *	Varicocelectomy Ivanissevich technique	Varicocelectomy subinguinal sclerotherapy	155
Abdel-Meguid *et al.* (2011)	Microsurgical subinguinal varicocelectomy	Subinguinal varicocelectomy	150
Mangoli *et al.* (2019)	Intracytoplasmic morphologically selected sperm injection	Intracytoplasmic sperm injection	150
Guo *et al.* (2017)	Doppler ultrasound at laparoscopic varicocelectomy	Laparoscopic varicocelectomy	147
Pan *et al.* (2016) *	Dietary supplement, Chinese herbal medicine and zinc selenium	Chinese herbal medicine	147
Ketabchi and Salajegheh (2018) *	Microscopic varicocelectomy and acupuncture	Sham acupuncture	140
Gopinath *et al.* (2013) *	Antioxidants	Placebo	138
Ketabchi *et al.* (2018)	Microsurgical subinguinal varicocelectomy	No intervention	138
Ghanaie *et al.* (2012)	Varicocele repair	No intervention	136
Colacurci *et al.* (2012)	FSH	Vitamin supplement	129
Haje and Naoom (2015) *	Tamoxifen and L-carnitine	Placebo	128
Yetkinel *et al.* (2019)	Microfluidic sperm selection	Conventional swim up technique	122
Yu *et al.* (2019) ^*^	Transcutaneous electrical acupuncture point stimulation 2 hertz	Lifestyle advice	121
El-Khayat *et al.* (2012)	Fallopian tube sperm perfusion	IUI	120
Figueira Rde *et al.* (2011)	Intracytoplasmic morphologically selected sperm injection	Intracytoplasmic sperm injection	120
Pan *et al.* (2013)	Inguinal varicocelectomy	Subinguinal varicocelectomy	120
Liu *et al.* (2020)	Green model lifestyle intervention	Conventional nursing	120
Salas-Huetos *et al.* (2021)	60 g mixed nuts	Nuts	119
Chen *et al.* (2020)	Yishen tongluo recipe	Minimally invasive surgery	116
Cyrus *et al.* (2015)	Varicocele and vitamin C	Varicocele and placebo	115
Amirzargar *et al.* (2012) *	Varicocelectomy and hCG	Varicocelectomy	113
De Geyter *et al.* (2019)	Sperm preparation and deselecting sperm with fragmented DNA	Conventional sperm preparation	111
Hosseini *et al.* (2016)	Ginger	Placebo	106
Selice *et al.* (2011)	FSH	No intervention	105
Busetto *et al.* (2018)	Nutritional supplement	Placebo	104
Azadi *et al.* (2011)	Varicocelectomy and zaditen	Varicocelectomy and placebo	103
Akin *et al.* (2014) *	Varicocelectomy and ligation with titanium clips	Varicocelectomy and ligation with surgical silk	100
Nasimi Doost Azgomi *et al.* (2018)	Withania somnifera	Pentoxifylline	100
Hafeez *et al.* (2011)	Herbal medicine	Allopathic medicine	100
Hou *et al.* (2015)	Microsurgical subinguinal varicocelectomy with testicular delivery	Microsurgical subinguinal varicocelectomy without testicular delivery	100
Parmegiani *et al.* (2012)	Physiological intracytoplasmic sperm injection	Intracytoplasmic sperm injection with sperm slow selection device	100
Pourmand *et al.* (2014)	Varicocelectomy	Varicocelectomy and l-Carnitine	100
Kızılay and Altay (2019)	Varicocelectomy and antioxidant	Varicocelectomy	93
Youssef and Abdalla (2015)	Single laparoscopic varicocelectomy	Transperitoneal varicocelectomy	93
ElSheikh *et al.* (2015) *	Vitamin E	Clomiphene citrate	90
Milardi *et al.* (2017) *	Prednisolone 5 mg	Prednisolone 12.5 mg	90
Peivandi *et al.* (2015)	Intrauterine insemination	Intrauterine insemination with fallopian tube sperm transfer	90
Rosety *et al.* (2017)	Exercise	No intervention	90
Wang *et al.* (2014)	Laparoscopic varicocelectomy	Transperitoneal varicocelectomy	90
Degirmenci *et al.* (2020) *	0–2 days sexual abstinence	2–3 days sexual abstinence; >4 days sexual abstinence	90
Qu *et al.* (2017)	Varicocelectomy and xuanju	Varicocelectomy	88
Bryniarski *et al.* (2017)	Laparoscopic varicocelectomy	Microsurgical varicocelectomy	84
Jin *et al.* (2016)	Intracytoplasmic sperm injection, selecting sperm bound to zona pellucida	Intracytoplasmic sperm injection	84
Raigani *et al.* (2014) *	Folic acid and zinc sulphate	Placebo	83
Farrag *et al.* (2015)	Recombinant FSH and intracytoplasmic sperm injection	Intracytoplasmic sperm injection	82
Lee *et al.* (2012)	Transperitoneal laparoscopic varicocele ligation	Laparoscopic single-site varicocele ligation	82
Kopets *et al.* (2020)	l-carnitine/acetyl-l-carnitine, l-arginine, glutathione, co-enzyme Q10, zinc, vitamin B9, vitamin B12, selenium	Placebo	83
Kang *et al.* (2013)	Varicocele ligation with vessel and lymphatic preservation	Varicocele ligation without vessel and lymphatic preservation	80
Kolahdooz *et al.* (2014)	*Nigella sativa* oil	Liquid paraffin	80
Park *et al.* (2016) *	Varicocelectomy and Chinese herbal medicine	Placebo	80
Tehrani *et al.* (2019) *	Hypo-osmotic swelling test and intracytoplasmic sperm injection	Intracytoplasmic sperm injection	80

* Multiarm trial.

### Primary and secondary outcomes

One hundred and four outcomes were reported across the included trials (Tables II and III).

**Table II:** Primary outcomes reported in the 100 largest randomized trials evaluating interventions for male infertility.

**Hormonal**
Serum oestradiol
Serum FSH
Serum LH
Serum sex hormone-binding globulin
Serum testosterone
**Metabolic**
Assessment of endothelial function
Bioelectrical impedance analysis
Blood pressure
BMI
Waist circumference
Serum markers of metabolic function
**Semen**
Semen pH
Semen volume
Sperm concentration
Sperm count
Sperm density
Sperm morphology
Sperm motility
Total motile sperm count
Sperm DNA fragmentation index
**Embryological**
Fertilization rate
Embryo development
Embryo quality
**Pregnancy outcomes**
Spontaneous pregnancy
Pregnancy following ART
Intrauterine pregnancy confirmed by ultrasound
Ongoing pregnancy confirmed by ultrasound (from 12 weeks onwards)
Ongoing pregnancy (>20 weeks)
Cumulative pregnancy rate
Live birth
Live birth at term
**Other**
Fertility awareness knowledge
Awareness of lifestyle factors affecting fertility
Satisfaction with sexual life
Sexual intercourse frequency
Patient-reported symptoms of androgen deficiency
Testicular pain

**Table III:** Secondary outcomes reported in the 100 largest randomized trials evaluating interventions for male infertility.

**Clinical examination**	**Pregnancy and childbirth**
Testicular volume	Gestational diabetes
Varicocele grade	Pre-eclampsia
Spermatic vein diameter	Stillbirth
Physical fitness assessed by continuous maximal incremental test	Gestational age at delivery
Bioelectrical impedance analysis	Live birth
Body mass index	Pregnancies to term
Waist circumference	Preterm birth
	Caesarean delivery
**Hormonal**	
Serum oestradiol	**Maternal complications**
Serum FSH	Anaemia requiring blood transfusion
Serum inhibin B	Haemolysis, elevated liver enzymes, low platelet count syndrome
Serum LH	Postpartum haemorrhage
Serum testosterone	Seizure
Serum inhibin B to FSH ratio	Sepsis
Prostate-specific antigen	
Haematocrit	**Neonatal outcomes**
Serum alanine aminotransferase	Birthweight
Serum aspartate aminotransferase	Small for gestational age
	Neonatal mortality
**Semen**	Bronchopulmonary dysplasia
Semen liquefaction time	Chromosomal anomalies
Semen pH	Necrotizing enterocolitis
Semen volume	Periventricular leucomalacia
Sperm concentration	Retinopathy of prematurity
Sperm count	Severe intraventricular haemorrhage
Sperm density	Structural malformations
Sperm morphology	
Sperm motility	**Intraoperative outcomes**
Sperm DNA fragmentation index	Operating time
Time to initiation of spermatogenesis	Number of internal spermatic veins ligated
Acrosome integrity	Number of internal spermatic arteries preserved
Sperm penetration assay	Haematoma formation
Levels of reactive oxygen species	Hydrocele
Malondialdehyde levels in seminal plasma	Infection
	Pain
**Embryological**	Pyrexia
Fertilization rate	Testicular atrophy
Number of embryos	
Embryo quality	**Postoperative outcomes**
Number of embryos available for transfer	Patient satisfaction
Number of embryos cryopreserved	Time to return to normal activities
Number of euploid embryos	Recurrence of varicocele
Number of blastocysts	
Blastocyst quality	**Resource utilization**
	Length of hospital stay
	Cost
**Early pregnancy**	
Spontaneous pregnancy	**Other**
Pregnancy following ART	Testosterone deficiency symptoms
βhCG detected pregnancy	Prostatic symptoms
Intrauterine pregnancy confirmed by ultrasound	Sexual dysfunction
Singleton pregnancy	
Multiple pregnancy	
Early pregnancy loss	
Ectopic pregnancy	
Late pregnancy loss	
Time to conception	
Cumulative pregnancy rate	

Thirty-six different primary outcomes were reported by 47 trials with 13 of these 47 trials (28%) reporting a definition of these outcomes. Commonly reported primary outcomes included semen quality (16 trials; 34%), pregnancy rate (13 trials; 28%) and live birth (4 trials; 9%).

Ninety-six trials reported 89 different secondary outcomes. Reported secondary outcomes were heterogeneous and included semen quality (52 trials; 54%), pregnancy rate (39 trials; 41%), pregnancy loss (9 trials, 10%) and live birth (9 trials; 10%) (Table III). Primary and secondary outcomes reported by the 25 largest RCTs are outlined in Table IV.

**Table IV:** Detailed primary and secondary outcomes reported in the largest 25 randomized trials revaluating interventions for male infertility.

		Primary outcomes										Secondary outcomes																							
Study	No. of participants	Serum testosterone levels	Semen analysis*	Sperm DNA fragmentation index	Fertilization rate	Embryo development	Spontaneous pregnancy rate	Pregnancy following ART	Ongoing pregnancy confirmed by USS	Livebirth	Livebirth at term	Reproductive hormones	Semen analysis*	Malondialdehyde levels	Sperm DNA fragmentation index	Fertilization rate	No. of embryos	Embryo quality	No. of embryos cryopreserved	Spontaneous pregnancy	Pregnancy following ART	Implantation rate	βhCG detected pregnancy	Intrauterine pregnancy confirmed by USS	Multiple pregnancy	Early pregnancy loss	Ectopic pregnancy	Pregnancy rate	Cumulative pregnancy rate	Time to conception	Pregnancy outcomes*	Gestational age at delivery	Livebirth	Preterm birth	Testosterone deficiency symptoms
Miller *et al.* (2019)	2772										●													●		●								●	

Schisterman *et al.* (2020)	2370		●	●						●													●	●	●	●	●				●			●	

Worrilow *et al.* (2013)	802								●													●				●									

Huang *et al.* (2020)	769												●	●	●									●		●						●	●		

Kovacic *et al.* (2010)	647								●							●		●				●		●		●			●						

Hajizadeh Maleki and Tartibian (2017a)	556		●	●																													●		

Hajizadeh Maleki and Tartibian (2020)	441												●		●																		●		

Hajizadeh Maleki and Tartibian (2018)	430												●		●																		●		

Hajizadeh Maleki and Tartibian (2017b)	419												●		●																		●		

Hasanen *et al.* (2020)	413								●									●				●	●												

Sun *et al.*, (2018)	358						●						●																						

Ding *et al.* (2015)	354		●				●	●																											

Turhan *et al.* (2011)	341						●						●																						

De Vos *et al.* (2013)	340				●	●															●														

Chen *et al.* (2019)	337																											●		●					

Almekaty *et al.* (2019)	330		●																									●							

Jensen *et al.* (2018)	330		●									●																				●			
Zhao *et al.* (2019)	316											●	●		●					●															

Velaers *et al.* (2012)	290				●												●	●					●	●											

Hajizadeh Maleki *et al.* (2019)	283			●																															

Habous *et al.* (2018)	282	●																																	●

Romany *et al.* (2014)	263									●						●							●			●									

Safarinejad (2011)	260												●																						

Leandri *et al.* (2013)	255												●		●	●	●		●			●		●									●		

Safarinejad (2011)	254											●	●																						

● denotes if outcome included in trial. *Semen analysis includes: semen volume; sperm concentration; sperm motility; sperm morphology; sperm count; and total motile sperm count.

* Pregnancy outcomes includes: caesarean section; pre-eclampsia; gestational diabetes; gestational age at delivery; birth weight; small for gestational age; severe postpartum maternal morbidity (including postpartum haemorrhage, anaemia requiring transfusion, sepsis, seizure, HELLP [haemolysis, elevated level of liver enzymes, low platelet count] syndrome, and pre-eclampsia with pulmonary oedema), major neonatal complications (including structural malformations, chromosomal anomalies, bronchopulmonary dysplasia, necrotizing enterocolitis, severe intraventricular haemorrhage, periventricular leukomalacia, and retinopathy of prematurity), still-birth and neonatal death.

USS, ultrasound scan.

### Definitions

Nine trials defined live birth as a primary or secondary outcome in two different ways: birth >37 weeks’ gestation; and birth <37 weeks’ gestation. The remaining seven trials did not define this term.

Pregnancy rate was reported by 51 trials as either a primary or secondary outcome, with 12 different definitions used by 21 trials. The remaining 26 trials did not define pregnancy rate, and 4 definitions were unclear. Definitions varied greatly, from a threshold of serum hCG >25 IU/l and the presence of a gestational sac on ultrasound scan (USS) to a viable foetus on transvaginal USS (Table V).

**Table V:** Variation in outcome reporting definitions across the 100 largest trials evaluating interventions for male infertility.

**Semen parameters (n = 75)**
WHO 2010 criteria (n = 32)
WHO 1999 criteria (n = 18)
WHO 1992 criteria (n = 3)
WHO 1999 and 1992 criteria (n = 1)
WHO edition not specified (n = 3)
Kruger strict morphology test (n = 1)
Undefined (n = 17)
**Pregnancy (n = 51)**
Serum hCG
Positive hCG test (n = 1)
>25 IU/l (n = 1)
>50 IU/l (n = 1)
>60 IU/l (n = 1)
Serum hCG >25 IU/l and USS confirmation (n = 1)
**Ultrasound examination**
Presence of one or more gestational sacs (n = 5)
Presence of a gestational sac with or without foetal heartbeat (n = 1)
Presence of a gestational sac with foetal heartbeat (n = 2)
Presence of a gestational sac or foetal heartbeat (n = 1)
>1 embryo with a foetal heartbeat (n = 4)
Foetal heart beat (n = 2)
Presence of a gestational sac or histological assessment confirming PoC (n = 1).
Unclear (n = 4)
Undefined (n = 26)
**Live birth (n = 9)**
Birth >37 weeks gestation (n = 1)
Birth <37 weeks gestation (n = 1)
Undefined (n = 7)

Three outcomes were selected to demonstrate variation across studies in how common outcomes were defined differently. The outcomes selected for this were semen analysis, pregnancy and live birth.

PoC, product of conception; USS, ultrasound scan; WHO, World Health Organization.

Semen quality was reported by 75 trials as either a primary or secondary outcome and there was a comparatively higher level of consensus between trials. A total of 57/75 trials defined these by the World Health Organization (WHO) criteria, having used either WHO 2010 (n = 32), WHO 1999 (n = 18), WHO 1992 (n = 3), WHO 1999 and 1992 (n = 1), in three the WHO semen analysis edition was not specified. Of the remaining studies, one used the Kruger strict morphology (Ketabchi *et al.*, 2018) and the remaining 17 trials did not define this outcome (Table V).

Not all of the studies included in our review used the most up to date edition of the WHO criteria available when conducting their trial. Studies defining semen analysis parameters using WHO 1992 criteria were commenced in 2012 and 2006 and could have utilized the WHO 1999 criteria when conducting the trial (Selice *et al.*, 2011; Sikka *et al.*, 2015). A similar issue was identified with some studies defining semen analysis criteria using WHO 1999, where the trial was commenced in 2013 or 2016 after the introduction of WHO 2010 (Haje and Naoom, 2015; Hosseini *et al.*, 2016; Tehrani *et al.*, 2019). Although these trials may initially appear to use an outdated version of the WHO semen analysis manual, their design may have occurred prior to the publication of an updated WHO criteria. Deviation from the initial analysis plan, potentially using two different WHO criteria or favouring one criterion in particular, may have been considered a violation of the trial protocol. To achieve consistent outcome reporting, should these trials have used new WHO criteria, they may no longer be comparable to older trials evaluating similar interventions for male infertility.

## Discussion

This systematic evaluation of the literature of RCTs in male factor infertility identified a range of primary and secondary outcomes relevant to male, maternal and neonatal participants. Many trials omitted important information about the primary outcome of the trial and how this was defined. Of the 100 randomized trials included in our review, only 47 clearly stated a primary outcome in their methodology. This lack of clear outcome reporting is not uncommon and has been identified as a problem in other areas, including in IVF, neonatal and endometriosis trials (Hirsch *et al.*, 2016; Wilkinson *et al.*, 2016; Webbe *et al.*, 2020).

### Interpretation

RCTs can be challenging to undertake and expensive to conduct; as such there is an ethical imperative to conduct them to the highest possible standards (Macleod *et al.*, 2014). Less than half of the 100 largest trials included in our review reported a clearly defined primary outcome, which represents a lost opportunity to obtain further robust data to inform clinical decision-making. Where trials reported the same primary outcome, often different measurement tools and endpoints were used to define these, which precludes pooling data from these trials. Even trials with seemingly consistent primary outcome reporting and definitions are not without their limitations. We identified 57 trials using WHO semen analysis methods to report, with primary or secondary outcomes. Although WHO semen analysis is a robustly developed standard, there have now been six different editions, of which three were used in our identified trials, although the most up to date edition was used in the majority of trials at the time these they were conducted (World Health Organization, 1992, 1999, 2010). Furthermore, semen is highly variable, even within individuals, which furthers the argument that semen quality may, in itself, not be an informative primary endpoint (Oshio *et al.*, 2004; Castilla *et al.*, 2006). This is demonstrated in some of the trials included in our review, which showed that improved semen quality did not correlate with improved pregnancy outcomes. This finding challenges the assumption that improved pregnancy outcomes are always associated with improved semen quality and not achieved through other factors including the population under study and the intervention used. Once such example is Huang *et al.* (2020), who demonstrated that folic acid supplementation was only effective at improving semen quality and pregnancy outcomes in a subgroup of patients with the homozygous polymorphism of the MTHFR 677 gene, while all other MTHFR polymorphisms studied showed no effect. Variable response in semen quality and pregnancy outcome was demonstrated by Hajizadeh Maleki *et al.* (2020) who investigated high-intensity interval training, reporting both semen parameters and live births. Patients categorized as asthenozoospermic, asthenoteratozoospermic, oligospermic or oligoasthenozoospermic demonstrated significantly improved semen quality following their exercise regime. Analysis of pregnancy outcomes in these cohorts, however, did not reveal a significant increase in live births. Another trial included in this review (Haje and Naoom, 2015) reported the impact of tamoxifen and l-carnitine on semen parameters and pregnancy outcomes. Although semen parameters, including sperm count, sperm motility and sperm morphology, were found to be improved in men receiving tamoxifen or tamoxifen with l-carnitine compared to placebo or l-carnitine only, these improvements did not translate into a significant increase in pregnancy rate.

In addition to outcome selection, inconsistent outcome reporting may result from a lack of validated instruments or poorly defined endpoints. One example is the assessment of sperm DNA fragmentation, for which at least eight different methods are available, with variable results obtained based on the test used and the laboratory undertaking the assessment (Agarwal *et al.*, 2016a,b; Pacey, 2018). Despite the large number of trials published on male factor infertility and the range of primary and secondary outcomes reported on, this inconsistency fundamentally limits their clinical utility and value to inform decision-making and patient care. In addition to difficulties in pooling results of trials, a lack of agreed core outcomes presents challenges for researchers designing future trials when selecting the outcomes to report, further compounded when considering factors such as sample size, cost and time.

Our systematic review is the first to report on the primary and secondary outcomes reported in male factor infertility trials and the definitions used for the primary outcome. It builds on work undertaken in other areas of reproductive health to identify causes of subfertility and harmonize the way these data are reported (Duffy *et al.*, 2017a,b; Lee *et al.*, 2020; Turner *et al.*, 2020; Rimmer *et al.*, 2021). At present, there is no consensus on definitions to be used for outcomes relevant to male factor infertility. To address inconsistencies in outcome reporting across male and female infertility trials, an international working group of healthcare professionals and researchers have developed the Core Outcome Measures for Infertility Trials (COMMIT) initiative (Core Outcomes in Women's and Newborn Health Initiative, 2014). This initiative will develop stakeholder-driven development of core outcome sets relevant to clinicians, researchers, and patients and has developed a consensus strategy for reporting core outcomes and standardizing their definitions (Duffy *et al.*, 2020, 2021).

As no core outcome set for male fertility trials has been developed, it is therefore not surprising that we identified little consistency between outcome reporting and definitions used. This is further compounded by the nature of male infertility trials and the interventions studied. Trials reporting on exercise or dietary supplements to improve semen quality may not report the same outcomes as techniques to select sperm to be used in ART to achieve a pregnancy. Researchers planning future male infertility trials should consider either using a core outcome set for these trials or, in the absence of this, consider reporting outcomes and outcome measures previously used in the literature to improve pooling of data across trials.

This review highlights inconsistencies in outcome reporting across male infertility trials but can also be used to identify commonly reported outcomes, when designing future trials. Using previously reported outcomes in new trials when evaluating interventions for male infertility may allow data from these trials to be pooled and meta-analysed. The outcomes identified in this review can be used to develop a core outcome set following discussion by a group of multinational, multiprofessional stakeholders as has been done for general infertility trials (Duffy *et al.*, 2021). Development of this core outcome set would guide researchers in which core outcomes to report, allow data from several trials to be pooled better inform patient care and reduce research waste (Duffy *et al.*, 2017a,b). We plan to develop a core outcome set for future male infertility research using outcomes reported in this review, using a modified Delphi method and modified Nominal Group Technique to identify relevant outcomes, their measurement and definitions.

### Strength and limitations

Our review has several strengths. The comprehensive search strategy and methodological design gives us confidence in the results we have identified. Collecting outcomes reported by 100 trials means that the outcomes and definitions identified reflect a significant body of work and are representative of the field of male infertility trials. To avoid bias, abstract screening and data extraction were undertaken by two independent reviewers, utilizing a third to resolve any queries and reach a consensus. However, our review is not without its limitations. For example, owing to the large number of RCTs published in male factor infertility, we only included the 100 largest trials. This means smaller trials were excluded, and inclusion of the data in these trials may have altered the results obtained and the conclusions drawn. Many trials reported on outcomes but did not clearly state it was the primary outcome or base a sample size calculation on this; as such, they were not included as a primary outcome in our review, despite being reported on by the authors. We did not contact the authors to clarify the primary outcomes where it was not clearly stated or if the researchers extracting data were unsure. We were also unable to validate the quality of the outcomes reported, as there is no validated tool to do this. As our review assessed outcome reporting and the definitions of these outcomes, we did not undertake an integrity check of the trials included in our review. However, we did identify that the conduct of some of the included trials could have been improved upon. These include one trial which was not registered (Karimi *et al.*, 2020) and four that were retrospectively registered (Moslemi Mehni *et al.*, 2014; Youssef and Abdalla, 2015; Taiyeb *et al.*, 2017; Ketabchi *et al.*, 2018). One trial obtained ethics committee approval after trial registration and the proposed recruitment period (according to the clinical trial registration), although specific recruitment dates were not included in the manuscript (Ketabchi *et al.*, 2018). One study had an enrolment period that ended 8 months prior to submission of the manuscript but also reported live birth as an outcome (Majumdar and Majumdar, 2013).

The conduct and integrity of RCTs are central to their ability to produce robust high-quality evidence (Li *et al.*, 2020). This would be improved by the implementation of a core outcome set for future male infertility research and assessment of trial integrity when undertaking systematic reviews and meta-analysis.

## Conclusion

Randomized trials reporting on interventions for male factor infertility frequently omit a primary outcome and often report these outcomes differently. This hinders the utility of these trials in how their results can be combined to inform health care professionals’ clinical decision-making and improve patient outcomes. Developing a core outcome set for male infertility trials will help inform how primary outcome measures are selected and reported on and translate into meaningful improvements in patient care.

## Supplementary data

Supplementary data are available at *Human Reproduction Open* online.

## Data availability

The data underlying this article will be shared on reasonable request to the corresponding author.

## Authors’ roles

M.P.R., R.A.H., V.S. and J.M.N.D. undertook the searches, data extraction and drafted the manuscript. M.P.R., R.A.H., V.S., R.A.A., Y.B., R.P.B., S.K.S., A.P., B.P., R.T.M., A.P., M.v.W., C.M.F., C.N. and J.M.N.D. participated in data analysis and interpretation, preparation of the manuscript and critically revising the paper. C.M.F., C.N. and J.M.N.D. conceived the idea of the manuscript. All authors approved the final version of the manuscript.

## Funding

This work received no funding.

## Conflict of interest

A.P.—chairman of external scientific advisory committee of Cryos International Denmark ApS, member of the scientific advisory board for Cytoswim LDT and ExSeed Health. Guest lecture at the ‘Insights for Fertility Conference’. M.v.W.—holds a ZON-MW research grant.

## Supplementary Material

hoac010_Supplementary_Data
