# Supplementary material for: Outcome reporting across randomized controlled trials evaluating potential treatments for male infertility: a systematic review
Source: Hum Reprod Open. 2022 Mar 4;2022(2):hoac010. doi: 10.1093/hropen/hoac010 (PMC8982407; doi:10.1093/hropen/hoac010)
Supplement: hoac010_Supplementary_Data [file hoac010_supplementary_data.docx]

**Supplementary Data** Review protocol and search strategy.

**Review Question**

What are the primary outcomes and outcome measures used in randomised control trials evaluating potential treatments for male infertility in the last ten years.

**Objectives**

1. Identify outcomes, outcome measures and the consistency of the reporting of these outcomes in randomised control trials investigating interventions for male infertility published in the last ten years.
2. To develop a comprehensive list of primary and secondary outcomes and map the definitions of commonly reported outcomes.

**Search Strategy**

A systematic review of randomised trials evaluating interventions for male infertility will be undertaken by searching for trials listed within the Cochrane Register of Controlled Trials (CENTRAL) between January 2010 and July 2021.

CENTRAL will be used as it is updated by the Cochrane Collaboration through regular searches of Cumulative Index to Nursing and Allied Health Literature (CINAHL), EMBASE, MEDLINE and PsycINFO. The following MeSH terms were used to identify relevant trials.

Keywords CONTAINS "idiopathic asthenospermia" or "idiopathic oligozoospermia" or "Sperm" or "sperm DNA integrity" or "sperm damage" or "sperm quality" or "sperm parameters" or "oligoasthenozoospermia" or "Oligoasthenospermia" or "oligoasthenoteratozoospermia" or "oligospermia" or "oligozoospermia" or "asthenospermia" or "asthenozoospermia" or "azoospermia" or "Male" or "male subfertility" or "male factor" or "male factor infertility" or "male factor subfertility" or "male fertility" or "male immune subfertility" or "male infertility" or "varicocele" or "varicocoele" or "varicocele-embolization" or "varicocele ligation" or "varicoceleoutcome" or "varicocelectomized" or "varicocelectomy" or "varicocoele repair" or "varicocoelectomy" or "varicocolectomy" or "hypogonadism" or "hypogonadotrophic hypogonadism" or Title CONTAINS "male subfertility" or "male infertility" or "male fertility"

**Study selection criteria**

**Inclusion**

Randomised control trials evaluating potential interventions for male infertility will be included in this review. Any intervention will be considered and not limited to pharmacological or surgical interventions. Trials which do not have a clearly defined primary outcome will still be included in this review. Following abstract screening, should more than100 trials be identified, data extraction will be limited to the 100 largest trials, determined by number of participants enrolled. Where data has been duplicated e.g. in a conference abstract and peer reviewed publication, data will be extracted from the full paper only.

**Exclusion**

Any study which is not a randomised control trial will be excluded. Studies not published in English will be excluded from this review.

**Population under investigation**

Participants enrolled in a randomised control trials evaluating an intervention for male infertility.

**Interventions**

Any intervention for male infertility.

**Main outcome & measure of effect**

**Main outcome:** stated primary outcome and how this outcome is defined.

**Secondary outcomes:** other reported outcomes, either secondary or potential primary outcomes, not clearly defined as a primary outcome.

**Data extraction of data**

Data will be extracted independently by two reviewers and a consensus reached should there be a discrepancy. A third reviewers opinion will be sought to reach a consensus if required. Data extracted will include, trial authors, year of publication, a brief description of intervention(s), size of cohort enrolled in the trial, primary outcome, secondary outcomes(s) and definition of the primary outcome.

**Risk of bias assessment**

No risk of bias assessment will be undertaken as this review aims to report on outcome reporting only.

**Data analysis plan**

We will report the interventions used in randomised trials evaluating interventions for male infertility as well as clearly stated primary outcomes and secondary outcomes. Where a trial does not clearly state a primary outcome, outcomes from these trials will be reported as a secondary outcome. Outcome definitions for commonly reported outcomes will be reported including the definition of this outcome.
